# Influence of Lactic Acid Surface Modification of Cellulose Nanofibrils on the Properties of Cellulose Nanofibril Films and Cellulose Nanofibril–Poly(lactic acid) Composites

**DOI:** 10.3390/biom11091346

**Published:** 2021-09-11

**Authors:** Ruth Anayimi Lafia-Araga, Ronald Sabo, Omid Nabinejad, Laurent Matuana, Nicole Stark

**Affiliations:** 1 Department of Chemistry, School of Physical Sciences, Federal University of Technology, Minna, NG 920001, Nigeria; ruth.arage@futminna.edu.ng; 2 U.S. Department of Agriculture, Forest Service, Forest Products Laboratory, Madison, WI 53726, USA; ronald.sabo@usda.gov; 3 School of Packaging, Michigan State University, East Lansing, MI 48824, USA; omidnabinejad@gmail.com (O.N.); matuana@msu.edu (L.M.)

**Keywords:** cellulose nanofibrils, lactic acid esterification, barrier properties, poly(lactic acid) nanocomposites, water absorption

## Abstract

In this study, cellulose nanofibrils (CNFs) were modified by catalyzed lactic acid esterification in an aqueous medium with SnCl_2_ as a catalyst. Films were made from unmodified and lactic acid-modified CNF without a polymer matrix to evaluate the effectiveness of the modification. Ungrafted and lactic acid-grafted CNF was also compounded with poly(lactic acid) (PLA) to produce composites. Mechanical, water absorption, and barrier properties were evaluated for ungrafted CNF, lactic acid-grafted CNF films, and PLA/CNF composites to ascertain the effect of lactic acid modification on the properties of the films and nanocomposites. FTIR spectra of the modified CNF revealed the presence of carbonyl peaks at 1720 cm^−1^, suggesting that the esterification reaction was successful. Modification of CNF with LA improved the tensile modulus of the produced films but the tensile strength and elongation decreased. Additionally, films made from modified CNF had lower water absorption, as well as water vapor and oxygen permeability, relative to their counterparts with unmodified CNFs. The mechanical properties of PLA/CNF composites made from lactic acid-grafted CNFs did not significantly change with respect to the ungrafted CNF. However, the addition of lactic acid-grafted CNF to PLA improved the water vapor permeability relative to composites containing ungrafted CNF. Therefore, the esterification of CNFs in an aqueous medium may provide an environmentally benign way of modifying the surface chemistry of CNFs to improve the barrier properties of CNF films and PLA/CNF composites.

## 1. Introduction

Cellulose nanofibril (CNF) research has continued to attract extensive interest because of its excellent properties such as abundance, high mechanical properties, biodegradability, low density, etc. CNFs have been considered a promising reinforcing agent for polymer-based nanocomposite applications due to their high specific strength, modulus, and aspect ratio. However, reinforcing properties can only be achieved with the fibers being homogeneously dispersed in the polymer matrix. Due to their hydrophilic character and high aspect ratio, CNFs tend to agglomerate through hydrogen bonding [1]. Once dried, CNFs are difficult to disperse in hydrophobic polymer-based matrices [2]. Achieving nanoscale dispersion of cellulose nanomaterials (CNMs) in thermoplastic composites using melt-blending techniques is difficult, and challenges include drying the nanomaterials without losing their nanoscale structure and compounding dry CNMs into polymeric matrices.

Wet compounding, in which CNM aqueous suspensions are compounded with polymers and dried in a single processing step, is emerging as a potential solution to overcome these challenges [2,3,4,5]. During this process, compounding and drying occur simultaneously, and dispersion of unmodified cellulose nanomaterials in polymer-based matrices has been demonstrated [2,3,4,5]. Employing such a strategy enables the processing of CNMs in an aqueous suspension, which is how they are typically produced. This technique is also compatible with some modification strategies. However, the number of aqueous-based modifications schemes that result in hydrophobized CNMs is limited [6,7,8,9]. One aim of the research presented here is to develop a water-based modification strategy that is compatible with wet compounding.

Adding CNMs to a biopolymer such as poly(lactic acid) (PLA) has been investigated as one way to improve PLA properties. Property improvements of PLA/CNF composites have been achieved by blending and compatibilization with a second polymer component with properties that can contribute to improving the fiber dispersion through effective interaction at the interface. However, the surface chemistry of CNF predisposes it to poor thermal stability, hygroscopicity, and incompatibility with non-polar polymer matrices [10]. It is therefore also necessary to modify the surface of CNF for good dispersion and better performance. Among the chemical modification methods, esterification of the surface of CNF has been widely used. This technique introduces functional groups onto the cellulose surface by condensation of carboxylic acid, acid anhydrides, or acyl chlorides with a cellulosic alcohol group through esterification or creation on an ester bond (O-C=O) [11]. 

Most esterification reactions are carried out in an organic solvent medium [12,13,14,15]. Missoum et al. [16] reported a method for the surface esterification of CNF for improved hydrophobic properties by means of solvent exchange in ionic liquids using acetic, butyric, isobutyric, and hexanoic anhydrides. Results obtained from secondary ion mass spectroscopy (SIMS) proved that the chemical surface modification occurred only at the surface of the CNF. The authors reported an increase in contact angle in the grafted samples relative to the ungrafted counterparts. They inferred that the surface esterification was successful and had resulted in a more hydrophobic CNF surface. In another study, Tingaut et al. [17] utilized a heterogeneous catalytic method with a solvent exchange of CNF suspension from water to DMF. The authors observed different percent acetylations from 1.5% to 17%. The resultant grafted CNFs were compounded with PLA using a solvent casting method in chloroform. It was reported that CNF with an increasing percentage of acetylation provided more translucent nanocomposites with reduced hygroscopicity and improved thermal stability when compared to unmodified CNF. The authors concluded that these properties could be improved through accurate control of acetylation. Although these modifications were successful, solvent-based modifications may be expensive or harmful, underscoring the need for green/sustainable chemistry in CNF modification [18]. 

Some non-solvent methods for esterifying CNFs have been demonstrated. Rodionova et al. [19] conducted gas-phase esterification on CNF films using trifluoroacetic acid anhydride (TFAA) and acetic acid (AcOH) in differing ratios at different temperatures and time (1:2 and 2:1) at 22 °C and 40 °C for 30 min or 40 min. The authors reported an increase in the contact angle from 41.2° for an unmodified film to 71.2° for the esterified films. This gas-phase esterification seems to be an effective technique for surface modification of CNF films or CNF aggregates. It was observed that not all the OH groups of the CNF are available in this case. Therefore, only a slight influence was detected. More recently, Sethi et al. [6] prepared nano papers from lactic-acid-modified cellulose nanofibers through esterification using an excess of lactic acid monomers and a catalyst in an aqueous solution. The authors reported that the modified nano papers displayed improved performance under humid conditions. Tensile modulus also improved with modification, but there was a loss in tensile strength. In the investigation, ultrasonication and hot pressing at elevated temperatures (100 °C and 150 °C) were believed to have aided the success of the esterification procedure. In addition, esterification in an aqueous medium is accompanied by a kinetic limitation, being an equilibrium reaction [20]. Therefore, to achieve a high ester yield, a large excess of one of the reactants and a catalyst are typically used [21]. 

In this work, a water-based catalyzed esterification of CNFs was carried out to graft lactic acid onto the surface of CNF without hot pressing the samples at elevated temperatures. In addition, PLA/CNF composites were produced containing both grafted and ungrafted CNFs. The mechanical, water absorption, and barrier properties of the nanocellulose and nanocomposites films were investigated and are reported here. 

## 2. Materials and Methods

### 2.1. Materials

Bleached Eucalyptus Kraft pulp was used to make the CNFs. Reagent grade lactic acid (90%) and Tin II Chloride (98%) were supplied by Sigma Aldrich, USA. PLA pellets (Ingeo 4044D, NatureWorks, LLC, Minnetonka, MN, USA) were used as the matrix. 

### 2.2. CNF Production

CNF was produced by grinding 10 L of a 2% pulp suspension in a super mass colloider MKZA6-2 SuperMassColloider (Masuko Sangyo Co. Ltd., Saitama, Japan) at 1500 rpm. The disc gap was adjusted from 0 to −100 µm as the pulp was loaded until the instrument delivered a current of 2A. The disc gap was adjusted to maintain the 2A current throughout the grinding. The suspension was ground for a period of six hours at an energy consumption of 2180 kW.

### 2.3. Lactic Acid Modification of CNF

To prepare the modified CNF, lactic acid was added to CNF aqueous suspensions in a 1 L beaker with SnCl_2_ as a catalyst at different ratios, as shown in Table 1. The suspension was mixed thoroughly at 5000 rpm for 10 min in a high shear mixer (model number HSM100LCIT, Charles Ross & Son Company, Hauppauge, NY, USA) and sonicated with a Fisher Scientific 705 probe sonicator (Fischer Scientific, Hampton, NH, USA) for 1 hr. It was then placed in the oven at 100 °C for approximately 40 h. The grafted CNF suspension was washed by diluting to about 0.2% and centrifuged for three cycles at 13,000 rpm for 10 min until the rinsate contained no evidence of lactic acid, as determined by FTIR. After each centrifugation cycle, the suspension was made up to the 0.2% concentration before another cycle was carried out.

### 2.4. Production of CNF Films

Ungrafted and LA-grafted CNF suspensions were diluted to 0.2% and filtered using a 142 mm diameter Millipore pressure filtration setup through a 0.45 µm membrane filter supported by a Whatman filter paper (Fisher Scientific). The wet films were dried in a stack of blotting and wax paper with a 23 kg load placed on it at room temperature for 24 h. The blotting papers were changed several times until the moisture level in the wet film was reduced to a minimum to avoid wrinkling. The assembly was then placed in an oven at 60 °C for 8 h for complete drying.

### 2.5. Production of Nanocomposites

PLA pellets were cooled with liquid nitrogen and ground in a Wiley Mill with a 2 mm mesh. 147 g of PLA powder was then compounded with 150 g of either ungrafted or LA-grafted CNF aqueous suspensions with 2% solids content. The resulting compounds consisting of 98% PLA and 2% CNF or LA-CNF were again cryogenically ground and processed into injection molded composites or extruded composite films using a microprocessing system (DSM Xplore, DSM Research, Geleen, The Netherlands). Injection-molded composites were produced using a 15 mL co-rotating twin-screw extruder at a barrel temperature of 180 °C and a screw speed of 100 rpm. The molten compound was collected and molded into type V tensile test samples (ASTM D6389) at an injection pressure of 15 bar and an injection time of 10 secs. The mold temperature was kept at 60 °C. Composites films were also compounded using the microprocessing system at a barrel temperature of 190 °C and a screw speed of 50 rpm, then extruded through a slit die with dimensions of 35 mm by 0.4 mm and wound on a drum.

### 2.6. Sample Characterization

#### 2.6.1. Fourier Transform Infrared Spectroscopy

The surface chemistry of the LA-grafted and the ungrafted CNF samples were characterized by FTIR spectroscopy using a Thermo Nicolet iZ10 FTIR-ATR spectrometer (Thermo Scientific, Verona, WI, USA) in an Attenuated Total Reflectance mode. Spectra were taken for 64 scans in the range of 4000–600 cm^−1^ with a resolution of 4 cm^−1^.

#### 2.6.2. Water Absorption

Rectangular strips of dimensions 70.0 × 14.0 × 0.10 mm (length, width, and thickness) of the LA-grafted and ungrafted CNF films were dried in an oven at 60 °C overnight and weighed. The dried samples were immersed in reverse osmosis (RO) water at ambient conditions. At predetermined time intervals, samples were removed, the excess water blotted out with lint-free wipes, and weighed. It was ensured that samples were weighed within 20 to 30 s of removal from the water. Water absorption was determined by measuring the increase in mass as a percentage of dry mass. Three specimens were tested for each sample, and the average results were recorded. 

#### 2.6.3. Water Vapor Permeability

CNF and PLA/CNF composite films were conditioned at 21.1 °C and 50% relative humidity (RH) for two weeks prior to conducting water vapor transmission tests, and all testing was performed at this condition. Water vapor transmission was determined gravimetrically, following the desiccant method in ASTM E96 with 68-3000 EZ-Cup vapometer assemblies (Thwing-Albert Instrument Company, West Berlin, NJ, USA). Samples were measured in five locations for thickness, and the average value was used. Desiccant (anhydrous calcium chloride) was placed in the base of the cup within 6 mm of the specimen. CNF film samples were cut into circles with a radius of 6.35 cm, sandwiched between two neoprene gaskets, and placed in the cup. An aluminum-threaded ring and Teflon seal secured the sample. Three replicates of either CNF or LA-CNF were tested. Water vapor transmission was also determined for the extruded composite films. In this case, the extruded films were narrower than the exposure area of the cup, so a mask was created for those specimens. The mask was constructed by cutting a 1 cm × 5 cm rectangular hole out of two aluminum disks. The polymer films were sandwiched between the discs and sealed with vacuum grease. The assemblies were weighed periodically to establish the linear region for the curve of weight gain as a function of time. Two replicates for each extruded film were tested. The samples were removed after 35 days of exposure. Water vapor transmission rate (WVTR) was calculated based on the following:

(1) $$\mathrm{WVTR}=\frac{G}{t\times A}$$

where *G*/*t* is the slope of the fitted straight line of water vapor mass gain vs. time, and *A* is the area of the exposed film, which is equal to 31.7 cm^2^.

The film water vapor permeability (WVP) was calculated using the following:

(2) $$\mathrm{WVP}=\frac{\mathrm{WVTR}}{\left(R_{1}-R_{2}\right)\times S}\times l\;$$

where *S* is the saturation vapor pressure of water at the test temperature (2493 Pa), *R*_1_ is the fractional RH on the vapor source side (50%), *R*_2_ is the fractional RH on the sink side (0%), and *l* is the film thickness. 

#### 2.6.4. Oxygen Permeability

The oxygen transmission rate was determined for CNF and LA-CNF films using a MOCON 2/22L oxygen test apparatus (ASTM D3985). The sample thickness was recorded in 5 locations and averaged before mounting in holders with an exposure area of 50 cm^2^. An individual zero was collected at the beginning of the test, followed by 36 exposure cycles, each 60 min long. An instrument re-zero was collected after every 2 tests for 30 min. The cell temperature was 23 °C. Samples were exposed to three different relative humidities (0%, 50%, and 90%) sequentially. Both the test gas and carrier gas were humidified to these setpoints. Three replicates were run for CNF and LA-grafted CNF films. The oxygen transmission rate was recorded as the volume of oxygen through the film per unit surface area. The permeability was calculated as the oxygen transmission rate divided by the pressure difference across the film and multiplied by the film thickness. 

#### 2.6.5. Tensile Properties

Tensile tests were conducted on the ungrafted CNF, LA-grafted CNF films, and PLA/CNF composites according to ASTM D638, at a 50% relative humidity and temperature of 22 °C. CNF films were cut into an ASTM D638 type V sample with a die (Qualitest, Ft. Lauderdale, FL, USA). Testing was carried out using an Instron 5865 system (Instron Engineering Corp, MA, USA), equipped with a 500 N load cell. CNF samples were tested with a crosshead speed of 0.1 mm/min, and the nanocomposites were tested at a crosshead speed of 1 mm/min. A total of 10 specimens of the nanocomposites were tested. All samples were conditioned for at least one week.

#### 2.6.6. Scanning Electron Microscopy

Scanning electron microscopy images of CNFs were taken using a Zeiss LEO 1530 (Zeiss/LEO, Oberkochen, Germany) at the University of Wisconsin Material Research Science and Engineering Center in Madison, WI. First, CNF suspensions were diluted to 0.04% in water, and the diluted suspensions were dried on SEM stubs. Once dried, the CNFs were sputter coated with gold using a Denton Desk-1 sputter coater (Denton Vacuum, LLC, Moorestown, NJ, USA) operated for 2.5 min at 30 mA. The SEM was operated with an accelerating voltage of 3.0 kV, and images were taken from the secondary electron detector.

## 3. Results and Discussion

### 3.1. Scanning Electron Microscopy

**Figure 1:**
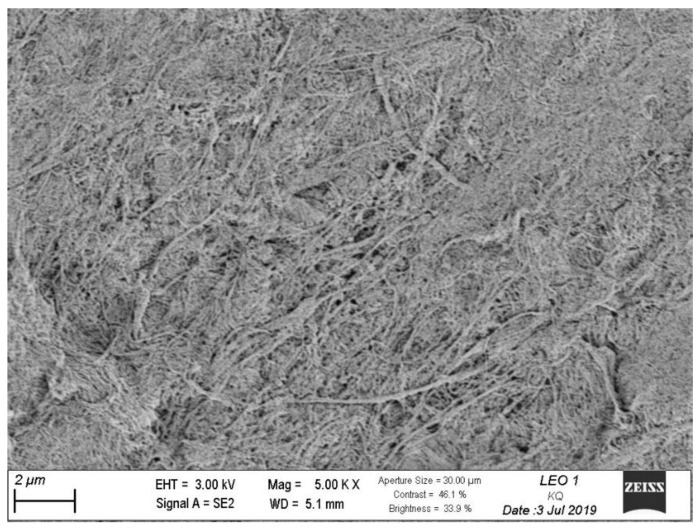
Scanning Electron Micrograph of CNF.

Figure 1 presents the morphology of the produced CNFs. As can be seen from the micrograph, there is a combination of nano- and microfibrils seen, as well as some larger fiber bundles, typical for mechanical grade CNFs. 

### 3.2. Esterification of CNF Samples

**Figure 2:**
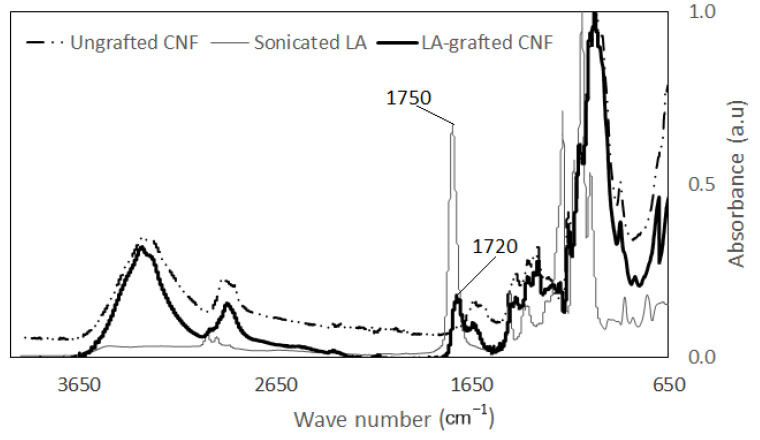
FTIR spectra of ungrafted CNF, LA-grafted CNF, and sonicated LA.

**Figure 3:**
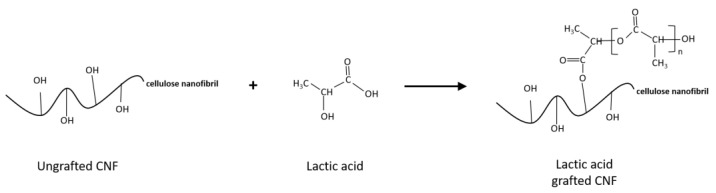
Schematic of lactic acid reacting with one hydroxyl group on the CNF surface through esterification, resulting in lactic acid-grafted CNF.

The effect of esterification on changes in the FTIR spectra of CNF and modified CNF using a 1:20 CNF:LA ratio is presented in Figure 2. Bands characteristics of cellulose can be seen in the spectra of the ungrafted and the LA-grafted CNF. Ester carbonyl groups appearing at 1750 cm^−1^ on the lactic acid are also seen to have shifted to 1720 cm^−1^ on the LA-grafted CNF. This could mean that the peak is in a different environment, possibly, on the surface of the CNF, and indicates that the esterification reaction is successful, as shown in Figure 2 [22,23]. Figure 3 presents a schematic of a lactic acid monomer reacting with one hydroxyl group at the surface of the CNF through esterification as proposed by Sethi et. al [6]. In this schematic there is one lactic acid monomer group attached at the surface of the CNF when n = 1, but it is possible that lactic acid monomers continue to polymerize the surface resulting in n>1. Because both the original grafting onto CNFs and further polymerization result in an ester group formation further work is needed to determine if a portion of the ester groups appearing on the FTIR spectra are also due to polymerization. The peak appearing at the 3300 cm^−1^ band is attributed to the OH stretching of the free OH groups on the cellulose. In addition, peaks at 1630 cm^−1^ may be attributed to absorbed water molecules on the ungrafted CNF. This peak is not apparent on the LA-grafted CNF spectra, suggesting may have resulted from a partial replacement of the free OH groups by the less polar ester groups [24,25]. 

**Figure 4:**
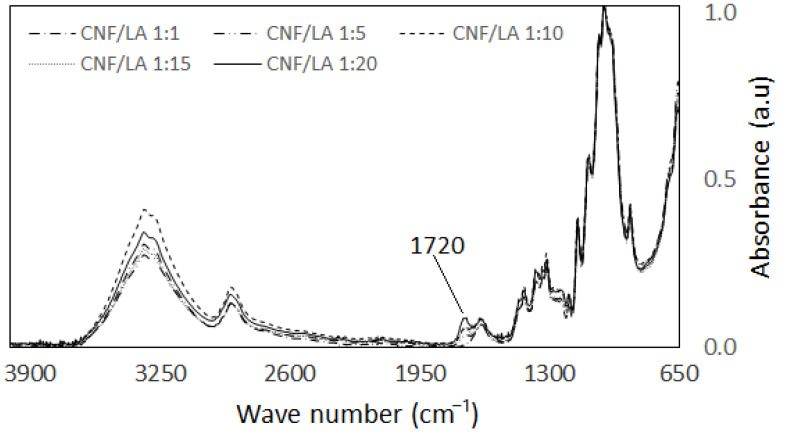
FTIR spectra showing the effect of the ratio of CNF:LA on the modification of CNF.

Figure 4 presents the effect of the CNF:LA ratio used during modification on the formation of ester groups (Table 1). There was no apparent peak at 1720 cm^−1^ for the spectra of CNF:LA ratio of 1:1, suggesting esterification did not occur. At higher LA content (i.e., CNF:LA ratios of 1:5, 1:10, and 1:15), the carbonyl peak at 1720 cm-1 was prevalent and of similar height. The highest LA content we investigated (i.e., CNF:LA of 1:20) showed the largest carbonyl peak at 1720 cm^−1^. This may indicate that the reaction between the CNF and the LA is more effective at higher LA content. This result agrees with the findings of Ramírez et al. [26]. Delgado et al. [27] conducted a kinetic study of an ion-exchange catalyzed esterification of lactic acid with ethanol to form ethyl acetate. It was observed that the equilibrium conversion of 9% was obtained for an ethanol-to-lactic acid molar ratio of 1 and increased up to a value of 35% as this ratio increased to 6. The authors noted that by using a large excess of one of the reactants, in this case, the lactic acid, the yield of ethyl acetate was enhanced. 

### 3.3. Tensile Properties

Table 2 summarizes the tensile properties of LA-grafted and ungrafted CNF films. The LA-grafted and ungrafted CNF films exhibited tensile modulus values of 16.4 and 13.5 GPa, respectively. This represents an increase of about 20% in the LA-grafted sample, relative to the ungrafted CNF, and could be attributed to increased stress transfer between the nanofibers. From the proposed reaction scheme (Figure 2), the hydroxyl groups from cellulose participated in the reaction. This should result in a strong covalent bond, making the entire structure become a rigid hybrid network and could be responsible for restricting the movement of CNFs when an external load is applied [6]. 

Contrarily, the tensile strength of the ungrafted CNF was found to be higher (131.1 MPa) than the LA-grafted sample (116.1 MPa). A 15% reduction in tensile strength and about 50% reduction in elongation at break were observed in the LA-grafted CNF when compared to the ungrafted sample. Replacement of the hydroxyl groups on the CNF surface by esterification may have broken down the hydrogen bonding within the CNF structure, resulting in premature failure under tension and consequently and lower tensile strength. This observation is consistent with the findings of Henrikson et al. (2008), who noted that interfibrillar bonds in CNF may be weakened by reduced hydrogen bonding [28]. Additionally, Sethi et al. [6] reported a 40% improvement in modulus, a 40% decline in the tensile strength, and about 80% reduction in the elongation at break in lactic-acid-esterified cellulose nano paper. It has been suggested that the energy provided by fiber–fiber bond in the paper is a factor that determines its strength properties. This energy is estimated to be approximately the sum of the hydrogen bonding and the energy friction between fibers when paper sheets are torn [29]. Therefore, any reduction in hydrogen bonding should, in principle, lower the tensile strength of the CNF.

The tensile properties of the nanocomposites are listed in Table 3. The presence of 2% CNF, modified or unmodified, did not significantly affect the mechanical properties of PLA. Robles et al. [30] reported a drop in tensile properties in PLA/CNF composites containing 2% CNF treated with 3-aminopropyl triethoxysilane relative to the untreated nanocomposites. The authors argued that at this filler content, the anisotropic characteristics of fillers in polymeric matrices play a major role in the response of the composites to uniaxial stress. This is said to generate more deformations in the composites and could lead to lower tensile properties. Here, we maintained the tensile properties while improving other properties, such as vapor permeability, as discussed below.

### 3.4. Water Absorption Properties

**Figure 5:**
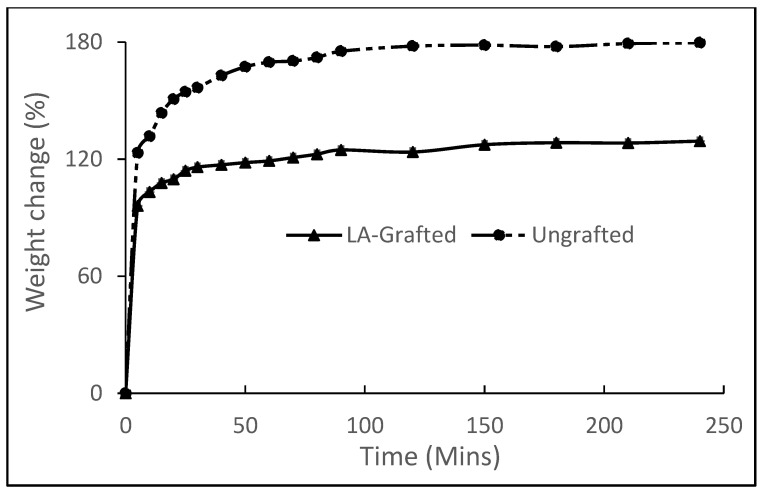
Water absorption properties of CNF films.

The water absorption properties of LA-grafted and ungrafted CNF films are shown in Figure 5, in which a rapid increase in the water uptake for both samples was observed in the first 5 min of immersion. This water uptake slowed during the next 30 min, and then a much slower water uptake occurred till the samples became substantially saturated. The ungrafted CNF displayed a higher water absorption than the grafted samples throughout the experiment. This unmodified sample recorded over 100% weight gain in the first 5 min and consistently maintained a faster water uptake throughout the course of the experiment. The LA-grafted CNF film displayed a better resistance to water absorption, with a 39% decrease in water uptake. This indicates that the lactic acid esterification significantly reduced the water absorption potentials of the LA-grafted CNF. This result agrees with the findings of Sethi et al. [6], who observed a 35% reduction in water absorption of lactic acid modified nano paper, relative to the unmodified samples, because of the reduced hydroxyl groups on the CNF. CNFs are highly hygroscopic, as water tends to displace the hydrogen bonds between nanofibrils of CNF films by substituting them with the water-nanofibril bonds, resulting in considerable absorption of water in the CNF [31,32]. In the LA-grafted sample, therefore, reduction in the hydroxyl groups on the surface of the CNF and the presence of an aliphatic carbon chain from lactic acid will deter the formation of the water–nanofibril bonds, leading to reduced moisture uptake. Reduced water uptake is particularly vital because resistance water is important in many applications, including packaging materials [31].

### 3.5. Barrier Properties

The impact of lactic acid esterification of CNF on the water vapor permeability of CNF and LA-grated CNF films is presented in Table 4. As with the water absorption behavior, the water vapor permeability (WVP) of the LA-grafted CNF films was lower (improved), compared to the ungrafted CNF sample. This is due to the hydrophilic nature of the ungrafted CNF. Cellulose materials have been reported to be inherently sensitive to the presence of gaseous and liquid water. This is a limiting factor when considering CNF in water vapor barrier applications. However, a reduction of 22% in WVP was observed in the LA-grafted sample. This decreased WVP is likely to increased hydrophobization of the CNF due to the three-carbon aliphatic chain of LA-grafted on the CNF. Rodionova et al. [33] studied the effect of acetylation on WVTR of MFC films and reported a reduction in WVTR from 234 g/m^2^/day to 167 g/m^2^/day within the first 30 min to 1 h of the acetylation. The authors attributed the decrease to the reduced solubility of water in the amorphous regions of the MFC structure as a result of the increasing fraction of acetylated hydrogen bonds that gradually prevented the amorphous parts of the MFC from absorbing water. In another study, which explored modified CNF films using high-temperature treatment between 100 °C and 175 °C [34], a reduction in the WVTR of thermally treated samples up to half of the untreated CNF was reported. The authors suggested that the improvement in WVTR was a result of an increase in hydrophobicity of the material arising from the internal hydrogen bonding of the free –OH groups, causing increased hydrophobicity.

The water vapor permeability of the nanocomposites films is shown in Table 5. The incorporation of ungrafted CNF did not improve the water vapor permeability of PLA. The barrier properties of the PLA/LA-grafted CNF composites were improved, compared with both the extruded PLA films and PLA/ungrafted CNF films, with an improvement of around 40% in comparison with the PLA/ungrafted CNF composites. This is expected, as the esterification of the CNF rendered it more hydrophobic by the addition of a C_3_ aliphatic chain through the ester bonds (Figure 3), thereby making it difficult for water vapor to penetrate. 

The impact of lactic acid esterification of CNF on the oxygen permeability (OP) of CNF and LA-grated CNF films is presented in Table 6. The oxygen permeability was determined at 0%, 50%, and 90% relative humidity. For all films, the oxygen permeability at 0% relative humidity was essentially zero. The oxygen permeability for all films was higher at 90% RH, compared with 50% RH, which is expected for hydrophilic CNFs. Modification with LA resulted in an 18% decrease in oxygen permeability at 50% RH and a 26% decrease in oxygen permeability at 90% RH. 

## 4. Conclusions

Surface esterification of CNF was carried out through lactic acid grafting in an aqueous medium. Ester carbonyl peaks were observed in the FTIR spectra of LA-grafted CNF, an indication that the esterification was successful. Tensile modulus of the grafted CNF films was found to be higher than that of the ungrafted sample, while the tensile strength and elongation at break reduced in the grafted CNF when compared to the ungrafted counterpart. LA-grafted CNF samples exhibited lower water absorption than the ungrafted CNF. Similarly, water vapor and oxygen permeabilities were reduced in the grafted samples, compared to the ungrafted ones. Improved water vapor permeability was also observed in the PLA/CNF composites containing LA-grafted CNFs. The water-based esterification of CNF presented in this research is an environmentally friendly strategy that may lead to workable CNF surface modifications and aid in the development of sustainable applications, such as packaging materials.

## Figures and Tables

**Table 1:** Weights of lactic acid, 0.4% CNF suspension, and SnCl_2_ added to a 1 L beaker to prepare CNF-LA- from various CNF-LA ratios based on 1.5 g total solid content of CNF.

CNF:LA Ratio	Lactic Acid (g)	CNF Suspension (g)	SnCl_2_ (µg)
1:1	1.5	375.0	**100**
1:5	7.5	375.0	**100**
1:10	15.0	375.0	**100**
1:15	22.5	375.0	**100**
1:20	30.0	375.0	**100**

**Table 2:** Tensile properties of LA-grafted and ungrafted CNF films.

Sample	Tensile Modulus (GPa)	Tensile Strength (MPa)	Elongation at Break (%)
Ungrafted CNF	13.5 ± 0.3	131.1 ± 4.9	7.28 ± 0.03
LA-grafted CNF	16.4 ± 0.7	116.1 ± 3.4	3.36 ± 0.01

**Table 3:** Tensile properties of PLA nanocomposites containing 2% ungrafted CNF or 2% LA-grafted CNF.

Sample	Tensile Modulus (GPa)	Tensile Strength (MPa)	Elongation at Break (%)
PLA Control	2.5 ± 0.2	57.6 ± 3.0	2.930 ± 0.004
PLA/Ungrafted CNF composites	2.6 ± 0.1	55.5 ± 1.7	2.960 ± 0.005
PLA/LA-grafted CNF composites	2.6 ± 0.2	58.5 ± 2.2	2.870 ± 0.003

**Table 4:** Water vapor permeability of CNF and lactic acid-grafted CNF films.

Sample	Water Vapor Permeability (g m/s m^2^ Pa) (×10^−11^)
Ungrafted CNF	3.77 ± 0.27
LA-grafted CNF	3.09 ± 0.31

**Table 5:** Water vapor permeability PLA composite films containing either 2% ungrafted CNF or 2% LA-grafted CNF.

Sample	Water Vapor Permeability (g m/s m^2^ Pa) (×10^−11^)
PLA Control	2.60 ± 0.13
PLA/Ungrafted CNF composites	2.74 ± 1.34
PLA/LA-grafted CNF composites	2.08 ± 0.22

**Table 6:** Oxygen permeability of CNF and lactic-acid grafted CNF films.

Sample	Oxygen Permeability at 50% RH (cc mm)/(m^2^ day atm)	Oxygen Permeability at 90% RH (cc mm)/(m^2^ day atm)
Ungrafted CNF	0.0210 ± 0.0030	3.148 ± 0.40
LA-grafted CNF	0.0172 ± 0.0007	2.319 ± 0.18

## Data Availability

The data presented in this study are available on request from the corresponding author.
